# Nucleolin mediated pro‐angiogenic role of Hydroxysafflor Yellow A in ischaemic cardiac dysfunction: Post‐transcriptional regulation of VEGF‐A and MMP‐9

**DOI:** 10.1111/jcmm.13552

**Published:** 2018-03-07

**Authors:** Jiang Zou, Nian Wang, Manting Liu, Yongping Bai, Hao Wang, Ke Liu, Huali Zhang, Xianzhong Xiao, Kangkai Wang

**Affiliations:** ^1^ Department of Pathophysiology Xiangya School of Medicine Central South University Changsha China; ^2^ Translational Medicine Center of Sepsis Key Lab of Hunan Province Central South University Changsha China; ^3^ Department of Geriatric Medicine Xiangya Hospital Central South University Changsha China; ^4^ Department of Laboratory Animals Xiangya School of Medicine Central South University Changsha China

**Keywords:** angiogenesis, Hydroxysafflor Yellow A, ischaemic cardiac dysfunction, MMP‐9, nucleolin, VEGF‐A

## Abstract

Hydroxysafflor Yellow A (HSYA), a most representative ingredient of Carthamus tinctorius L., had long been used in treating ischaemic cardiovascular diseases in China and exhibited prominently anticoagulant and pro‐angiogenic activities, but the underlying mechanisms remained largely unknown. This study aimed to further elucidate the pro‐angiogenic effect and mechanism of HSYA on ischaemic cardiac dysfunction. A C57 mouse model of acute myocardial infarction (AMI) was firstly established, and 25 mg/kg HSYA was intraperitoneally injected immediately after operation and given once, respectively, each morning and evening for 2 weeks. It was found that HSYA significantly improved ischaemia‐induced cardiac haemodynamics, enhanced the survival rate, alleviated the myocardial injury and increased the expressions of CD31, vascular endothelial growth factor‐A (VEGF‐A) and nucleolin in the ischaemic myocardium. In addition, HSYA promoted the migration and tube formation of human umbilical vein endothelial cells (HUVECs), enhanced the expressions of nucleolin, VEGF‐A and matrix metalloproteinase‐9 (MMP‐9) in a dose‐ and time‐dependent manner. However, down‐regulation of nucleolin expression sharply abrogated the effect mentioned above of HSYA. Further protein‐RNA coimmunoprecipitation and immunoprecipitation‐RT‐PCR assay showed that nucleolin binded to VEGF‐A and MMP‐9 mRNA and overexpression of nucleolin up‐regulated the mRNA expressions of VEGF‐A and MMP‐9 in the HUVECs through enhancing the stability of VEGF‐A and MMP‐9 mRNA. Furthermore, HSYA increased the mRNA expressions of VEGF‐A and MMP‐9 in the extract of antinucleolin antibody‐precipitated protein from the heart of AMI mice. Our data revealed that nucleolin mediated the pro‐angiogenic effect of HSYA through post‐transcriptional regulation of VEGF‐A and MMP‐9 expression, which contributed to the protective effect of HSYA on ischaemic cardiac dysfunction.

## 1 INTRODUCTION

Coronary artery heart disease has become the leading cause of death and disability globally in the last 15 years,^1^ and it is mainly attributed to coronary artery stenosis or occlusion‐induced myocardial hypoperfusion or ischaemia, which can result in acute myocardial infarction (AMI), myocardial fibrosis and further cardiac dysfunction. Persistent myocardial ischaemia can subsequently result in irreversible myocardial injury, and the injured myocardial cells are very difficult to regenerate. Consequently, the myocardium supplied by occluded coronary artery becomes disable and even life‐threatening.^2^, ^3^ Therefore, more and more attention has been focused on the therapeutic angiogenesis.

Angiogenesis, an essential event involved in various physiological and pathological processes, such as wound healing, formation of granulation tissue and embryogenesis, promotes the growth of new capillary blood vessels and restores the blood flow of ischaemic tissue.^4^, ^5^, ^6^ It has been proved that neoangiogenesis can effectively restore the blood perfusion of coronary artery and further contribute to the myocardial regeneration.^4^, ^5^, ^6^ Nowadays, therapeutic neovascularization or neoangiogenesis has been increasingly considered as a promising therapeutic strategy for ischaemic heart diseases due to its effectiveness and invasive property, and more and more attention has been focused on searching for drugs with strong pro‐angiogenic activity and little toxicity.^7^, ^8^, ^9^

Hydroxysafflor Yellow A (HSYA), a most representative and water‐soluble ingredient of Carthamus tinctorius L., has long been used in the treatment of myocardial ischaemia in China and exhibits prominently antiplatelet, anti‐inflammatory, antioxidant and pro‐angiogenic activities.^10^, ^11^, ^12^, ^13^ What is worth to be mentioned is the pro‐angiogenic effect of HSYA which has been reported in different ischaemic models recently.^12^, ^14^ However, the underlying mechanisms have not yet been fully elucidated.

Nucleolin is a ubiquitously expressed and multifunctional DNA‐, RNA‐ and protein‐binding protein in the nucleolus of eukaryotic cells.^15^, ^16^ It is conserved in animals, plants and yeast and plays an essential role in various pathophysiological processes such as assembly of ribosomes, DNA and RNA metabolism, chromatin structure, rDNA transcription, rRNA maturation, nucleogenesis, cell proliferation and apoptosis**,** tumour growth and angiogenesis.^15^ The central region of nucleolin protein contains 4 tandem RNA‐binding domains, of which the first 2 determine the RNA‐binding specificity and affinity of nucleolin recognition element ((U/G)CCCG(A/G)). Nucleolin regulates the mRNA stability of target genes and consequently mediates their post‐transcriptional control through binding the nucleolin recognition element to corresponding binding elements of different target genes.^17^, ^18^, ^19^ It has been reported that nucleolin can mediate the expressions of angiogenesis‐related genes, including vascular endothelial growth factor‐A (VEGF‐A) and matrix metalloproteinase‐9 (MMP‐9).^14^, ^20^, ^21^ However, whether nucleolin mediates the pro‐angiogenic effect of HSYA through post‐transcriptional regulation of these angiogenesis‐related genes is largely unknown. In this study, the cardioprotective and pro‐angiogenic effect of HSYA on the ischaemic cardiac dysfunction were firstly determined, and then, the role as well as the underlying mechanisms of nucleolin in the pro‐angiogenic effect of HSYA was further investigated.

## 2 MATERIALS AND METHODS

### 2.1 Reagent

HSYA (CAS No. 78281‐02‐4) was obtained from Shanghai PureOne Biotechnology Co., Ltd (Shanghai, China). The antibodies to nucleolin, β‐actin and CD31 were purchased from Sigma‐Aldrich (St. Louis, MO, USA). The VEGF‐A and MMP‐9 ELISA kits were purchased from Boster Biological Technology, Ltd. (Wuhan, China).

### 2.2 Establishment and treatment of a mouse model of AMI

Male C57 mice weighed 20‐25 g were purchased from the Center of Experimental Animals in Central South University of China. The mice were kept on a 12‐h light/dark cycle and housed individually with free access to food and water throughout the experiment. Animal use procedures were approved by the animal welfare ethics committee of Central South University (protocol number 20151801). A mouse model of AMI was established by the occlusion of left anterior descending coronary artery (LAD). Firstly, during the inhalational anaesthesia with isoflurane, the electrocardiogram of mice was recorded. Secondly, under sterile conditions, the heart was exposed through a left thoracotomy in the fourth intercostal space, then the LAD was ligated, the thoracic incision was closed when the elevation of ST segment was found in the electrocardiogram.

The mice were randomly divided into 4 groups as follows: (1) sham‐operated group (sham); (2) sham‐operated group with HSYA treatment (sham + HSYA); (3) LAD‐occluded group (AMI group); (4) LAD‐occluded group with HSYA treatment (AMI + HSYA). Mice in the sham group and sham + HSYA group underwent thoracotomy without the ligation of LAD. HSYA was administrated immediately after the operation through intraperitoneal injection at 25 mg/kg, while mice in the sham group and AMI group were given equal volume of normal saline to HSYA. Then, 25 mg/kg HSYA and the equal volume of saline were given to the mice once, respectively, each morning and evening.

### 2.3 Measurement of cardiac haemodynamics

According to the protocol we previously reported,^22^ the performance of left ventricle (LV) was measured in mice under inhalational anaesthesia with isoflurane. A small cannula filled with heparin saline (500 U/mL) was inserted into the LV through the apex with chest open and mechanically ventilated and positioned along the cardiac longitudinal axis. After stabilization for 2 minutes, the pressure signal was continuously recorded using a MacLab A/D converter (AD Instruments, Mountain View, CA, USA). The left ventricular systolic pressure (LVSP) and left ventricular end‐diastolic pressure (LVEDP) were measured, and the maximal slope of systolic pressure increment (+d*p*/d*t*) and diastolic pressure decrement (−d*p*/d*t*) were calculated.

### 2.4 Measurement of myocardial infarct size

The myocardial infarct size was measured by 2% triphenyltetrazolium chloride (TTC) staining as described previously.^23^ Positive TTC staining was in red colour, and the infracted area was pale. Images were analysed by Image‐Pro Plus, and the infarct size was expressed as a percentage of ischaemic area at risk (% IAR).

### 2.5 Pathological examination

The HE staining was performed as described previously.^23^ Masson's trichrome staining was performed to display the fibration from viable myocardium in the peri‐infarct zone. All the pictures were photographed under an optical microscope (Olympus). The extent of collagen deposition was calculated by Image‐Pro Plus software (Media Cybernetics, Bethesda, MD, USA).

### 2.6 Immunohistochemical staining of CD31 and microvessel density determination

The expression of CD31 in the myocardium was detected by immunohistochemistry as described previously.^24^ Microvessel density (MVD) was calculated through identifying 3 fields from area of highest vascular density in the ischaemic myocardium per slide of each sample and counting at low power lens (magnification, 200×).

### 2.7 Cell culture

Human umbilical vein endothelial cells (HUVECs) were obtained from American Type Culture Collection and cultured in DMEM medium (Hyclone, USA) supplemented with 10% foetal bovine serum (Hyclone), 100 U/mL penicillin, 100 μg/mL streptomycin, 0.1 mg/mL heparin and 0.03 mg/mL endothelial cell growth supplements (Sigma, St. Luis, MO, USA) at 37°C under a humidified atmosphere of 95% air with 5% CO_2_ to allow the cells to grow and form a monolayer in the flask. After confluence, cells were trypsinized using 0.25% trypsin in Hanks buffer for 2 minutes and resuspended in complete culture medium.

### 2.8 Real‐time quantitative polymerase chain reaction

The total RNA of myocardial tissues and HUVECs were extracted by TRIzol and reverse‐transcribed to cDNA with PrimescriptTM RT reagent kit with gDNA; eraser according to the manufacturer's instructions (Takara shuzo Co., Kyoto, Japan). The concentration and purity of total RNA were determined by measuring the OD260 and OD260/OD280 ratio, respectively. The mRNA of VEGF‐A, nucleolin and MMP‐9 were measured by SYBR^®^ Premix Ex Taq™ (Takara shuzo Co.) through an ABI 7500 real‐time PCR system (Life Technology Corporation, Carlsbad, CA). Each cDNA sample was carried out in triplicate. The relative quantitation of mRNA was analysed using the equation as follows: Ratio = 2^−▵▵Ct^ and normalized by GAPDH. The following primers for mouse were used: VEGF‐A forward: 5′‐CTGCTGTAACGATGAAGCCCTG‐3′ and reverse: 5′‐GCTGTAGGAAGCTCATCTCTCC‐3′; nucleolin forward: 5′‐TGAGGGCAGAACAATCAGGCTG‐3′ and reverse: 5′‐GGTCTCTTCAGTGGTATCCTCAG‐3′; MMP‐9 forward: 5′‐GCTGACTACGATAAGGACGGCA‐3′ and reverse: 5′‐TAGTGGTGCAGGCAGAGTAGGA‐3′; GAPDH forward: 5′‐CATCACTGCCACCCAGAAGACTG‐3′ and reverse: 5′‐ATGCCAGTGAGCTTCCCGTTCAG‐3′. Primers for human were as follows: VEGF‐A forward: 5′‐TTGCCTTGCTGCTCTACCTCCA‐3′ and reverse: 5′‐GATGGCAGTAGCTGCGCTGATA‐3′; nucleolin forward: 5′‐GCCTGTCAAAGAAGCACCTGGA‐3′ and reverse: 5′‐GAAAGCCGTAGTCGGTTCTGTG‐3′; MMP‐9 forward: 5′‐ACGCAGACATCGTCATCCAG‐3′ and reverse: 5′‐ CAGGGACCACAACTCGTCAT‐3′; GAPDH forward: 5′‐AATGGGCAGCCGTTAGGAAA‐3′ and reverse: 5′‐GCGCCCAATACGACCAAATC‐3′.

### 2.9 Immuoblotting

Myocardial tissues and HUVECs were homogenized or scraped with the lysis buffer as previously described.^25^ Tissue homogenates and cell lysates were centrifuged at 4°C, 14 000 g for 15 minutes. The concentrations of protein in supernatant were determined by BCA protein assay kit. 20–50 μg protein was loaded to 10% SDS polyacrylamide gel electrophoresis and then transferred to polyvinylidene difluoride (PVDF) membranes (Millipore, USA). Enhanced or super chemiluminescence (Invitrogen, USA) was used to detect specific proteins according to the manufacture's instruction. The relative band intensity was quantified by Quantity One software.

### 2.10 Scratch wound‐healing assay

As we reported previously,^20^ the migration of HUVEC was determined by the scratch wound‐healing assay. Six‐well plates were seeded with cells to a final density of 2 × 10^5^ cells per well, and a 10‐μL sterile pipette tip was used to scratch at the mid of each well until the cells were adherent to the plate. Cells in each well were treated by 50 μg/mL HSYA or vehicle and photographed under a microscope at different time‐points. The area of wound edges was measured and compared between different time‐points. All scratch assays were performed in quadruplicate.

### 2.11 In vitro angiogenesis assays: tube formation on Matrigel

The tube formation assay was performed according to the manufacture's protocols of BD Matrigel Matrix (BD Biosciences, USA). For preparation, Matrigel Matrix was incubated and fully dissolved at 4°C overnight, and 50 μl Matrigel Matrix was applied to each well of 96‐well plates and then incubated at 37°C for 30 minutes. Then, 150 μl cell culture medium containing 3‐5 × 10^3^ HUVECs was seeded on the matrigel in each well. Cells in each well were treated by HSYA or vehicle for different time. The tubular formation of HUVECs was observed and photographed using an inverted phase‐contrast microscope in 3 random fields. Image analysis was performed by Wimasis WimTube software to calculate the number of tubules, loops and branch points.^20^

### 2.12 Determination of the interaction between nucleolin and MMP‐9, VEGF‐A mRNA

Protein‐RNA coimmunoprecipitation was performed to determine the interaction between nucleolin and MMP‐9, VEGF‐A mRNA as we reported previously.^18^, ^23^ 5 μg of nucleolin antibody was added into the pre‐cleared protein extract, followed by a period of 1 hour incubation at 4°C. Then, the mixture of nucleolin antibody and protein extract was mixed with pre‐washed 200 μL protein A/G magnetic beads at 4°C overnight. After being centrifugated at 4°C, 10 000 *g* for 30 seconds, the supernatant was removed, then total RNA was extracted from the magnetic beads and subjected to real‐time quantitative PCR to detect the mRNA expression of MMP‐9 and VEGF‐A.

### 2.13 Measurement of the stability of MMP‐9 and VEGF‐A mRNA

Human umbilical vein endothelial cells transfected with nucleolin‐specific siRNA (siRNA‐NCL), pCMV‐GFP‐nucleolin (pCMV‐NCL) and the corresponding siRNA‐scramble or control plasmid for 48 hours were incubated with either 0.5% ethanol or 5 mg/mL actinomycin D in 0.5% ethanol for 1, 3 and 6 hours, respectively. Then, the total RNAs were isolated and reverse‐transcribed into cDNA. Real‐time quantitative PCR of the cDNAs was performed to detect the mRNA expressions of MMP‐9 and VEGF‐A.

### 2.14 Enzyme‐linked immunosorbent assay

Enzyme‐linked immunosorbent assays were performed to detect the VEGF‐A and MMP‐9 contents according to the manufacture's protocols.

### 2.15 Statistical analysis

All data were analysed by SPSS 18.0 software. Measurement data were shown as mean ± SEM of 3 different experiments and analysed by unpaired 2‐tailed Student's *t* tests. Kaplan‐Meier analysis was performed to compare the differences in survival rate between different groups. *P* < .05 was considered statistically significant.

## 3 RESULTS

### 3.1 HSYA improved ischaemia‐induced cardiac haemodynamics and enhanced the survival rate of AMI mice

**Figure 1:**
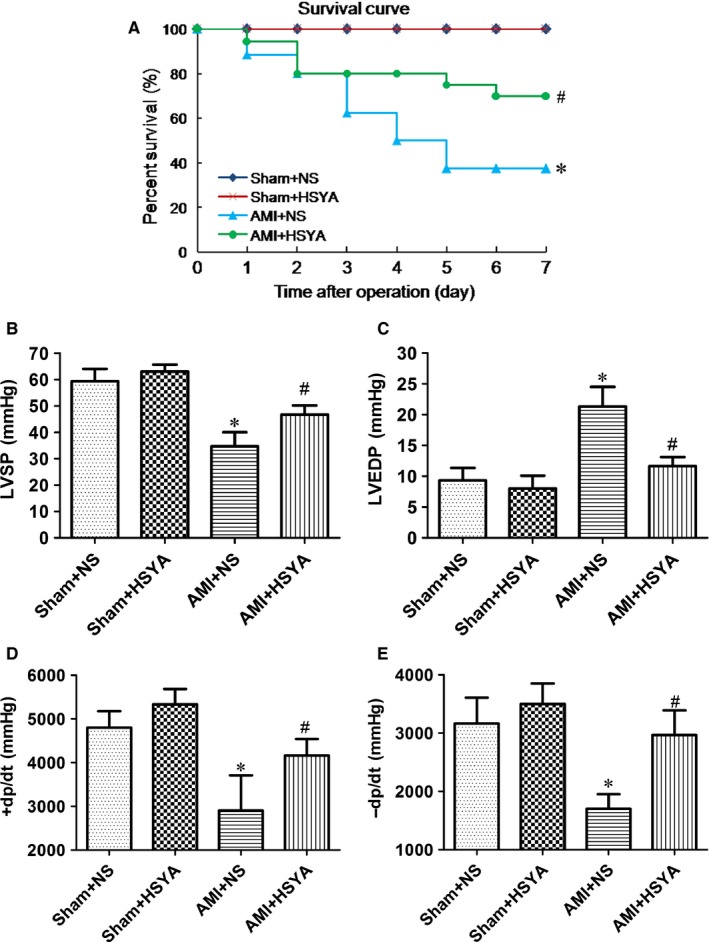
Hydroxysafflor Yellow A improved the ligation of left anterior descending coronary artery‐induced cardiac haemodynamics and enhanced the survival rate of AMI mice. A mouse model of AMI was established by the ligation of LAD. HSYA was administrated immediately after operation through intraperitoneal injection at 25 mg/kg, while mice in the sham group and AMI group were given equal volume of normal saline (NS) to HSYA. A, Kaplan‐Meier test was performed to analyse the survival of mice (n = 20 per group; *P <* *.05 vs Sham+NS, #*P *<* *.05 vs AMI + NS). B–E, The effect of HSYA on the ligation of LAD‐induced changes of LVSP, LVEDP, +d*p*/d*t*, −d*p*/d*t* of mice (n = 5 per group; *P <* *.05 vs Sham + NS, #*P *<* *.05 vs AMI + NS)

To investigate the protective effect of HSYA on AMI, the cardiac haemodynamics and survival of AMI mice were firstly analysed. As shown in Figure 1A, the survival rate of AMI mice was 37.5%, which was significantly lower than that of the sham‐operated controls (100%) (*P* < .05). However, HSYA treatment obviously elevated the survival rate of AMI mice to 70.6% (*P* < .05). Moreover, the haemodynamic indexes such as LVSP, +d*p*/d*t* and −d*p*/d*t* of saline‐treated AMI mice showed significant decline in comparison with the sham‐operated controls (*P* < .05, Figure 1B,D,E), while the LVEDP was evidently increased (*P* < .05, Figure 1C). However, HSYA treatment significantly abrogated the changes of LVSP, LVEDP, +d*p*/d*t* and −d*p*/d*t* of AMI mice (*P* < .05, Figure 1B–E). No significant difference was found in the haemodynamic indices and survival between sham‐operated controls treated by saline and HSYA (*P* > .05, Figure 1B–E). These in vivo results demonstrated that HSYA treatment could effectively mitigate ischaemia‐induced cardiac dysfunction and improve the survival of AMI mice.

### 3.2 HSYA diminished the myocardial infarction size and alleviated the myocardial injury

**Figure 2:**
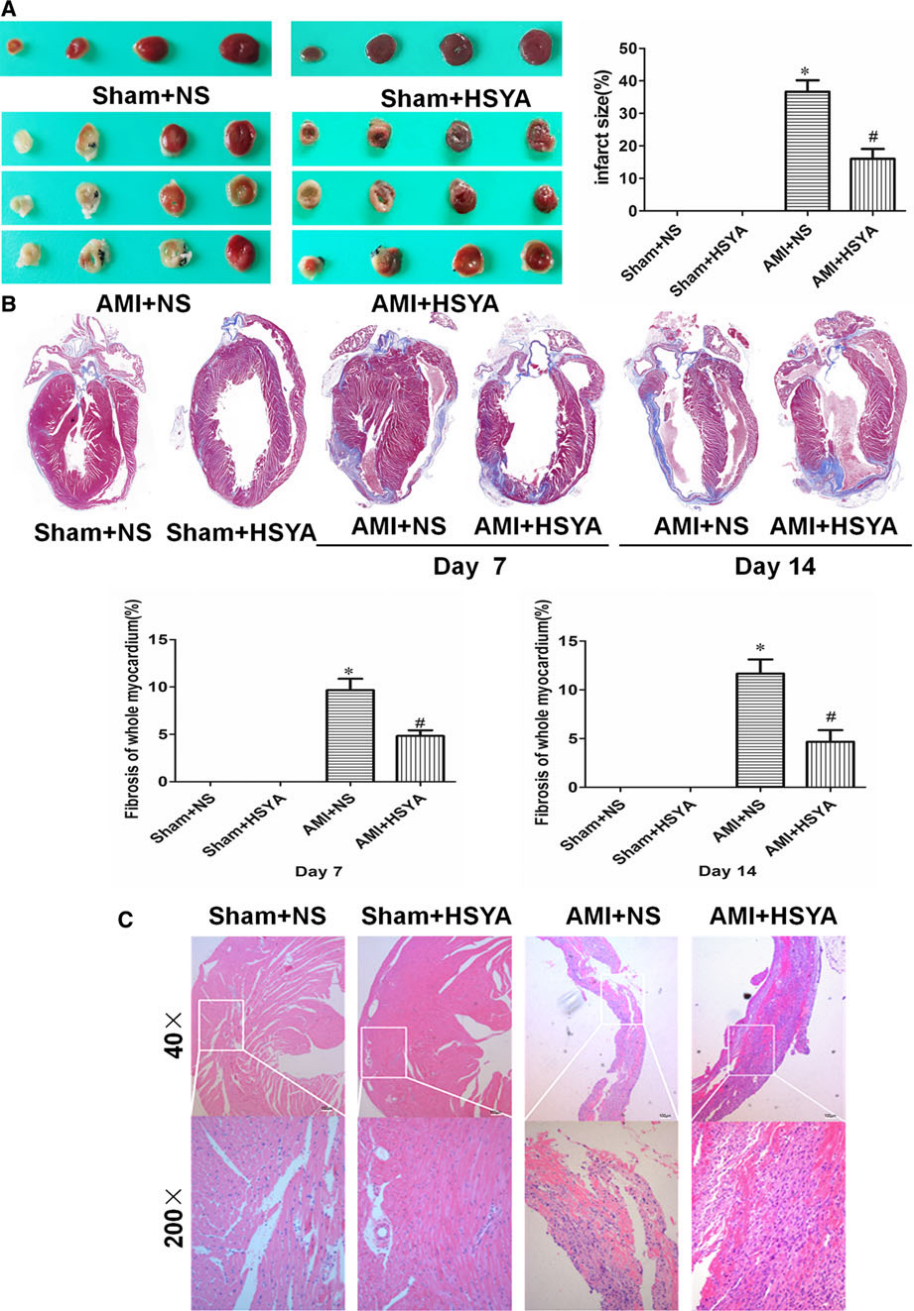
Hydroxysafflor Yellow A diminished the myocardial infarct size and alleviated the myocardial injury. (A) Representative images of TTC‐stained heart sections (left) and the statistics histogram of myocardial infarct size of mice with or without HSYA treatment for 7 days (right) (n = 5 per group; *P <* *.05 vs Sham+NS, # *P *<* *.05 vs AMI + NS). (B) Representative images of Masson's Trichrome‐stained mice heart sections (upper) and the fibrosis of whole myocardium subjected to ischaemia with or without HSYA treatment for 7 and 14 days (nether) (n = 5 per group; *P <* *.05 vs Sham + NS, # *P *<* *.05 vs AMI + NS). (C) Representative images of HE staining of heart sections subjected to ischaemia with or without HSYA treatment for 7 days (Scale bar = 100 μm)

To further determine the protective effect of HSYA on the myocardial ischaemic injury, TTC, Masson and HE staining were performed. It was shown that HSYA markedly attenuated LAD ligation‐induced myocardial infarct size in mice (*P* < .05, Figure 2A). Masson staining showed more and more collagen production on the 7th and 14th day after LAD ligation, while HSYA treatment alleviated the collagen deposition of AMI mice by 50.05% and 59.98% (*P* < .05, Figure 2B). Furthermore, AMI mice showed more overt left ventricular wall thinning and more serious myocardial fibrosis as compared with the sham‐operated controls, the myocardial cells were almost completely substituted by the fibroblasts, whereas a greater number of myocardial cells could be observed in HSYA‐treated AMI mice, suggesting that HSYA treatment could alleviate LAD ligation‐induced acute ischaemic myocardial injury (Figure 2C).

### 3.3 HSYA increased the angiogenesis and nucleolin expression in the ischaemic myocardium of AMI mice

**Figure 3:**
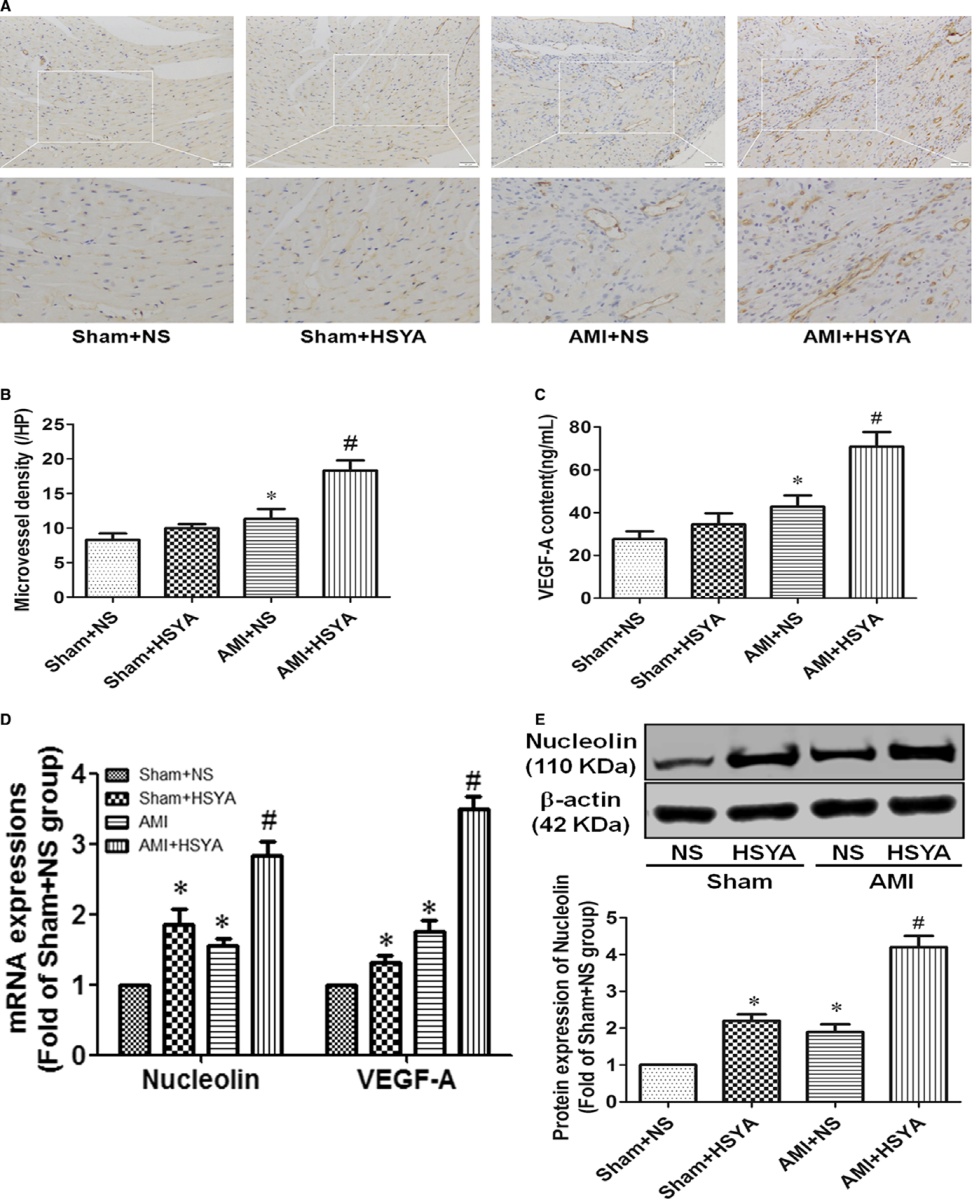
Hydroxysafflor Yellow A increased the angiogenesis and nucleolin expression in the ischaemic myocardium of AMI mice. (A) The expression of CD31 in the heart of mice was detected by immunohistochemistry (Scale bar = 100 μm). (B) Microvessel density (MVD) was calculated through identifying 3 fields from area of highest vascular density in the ischaemic myocardium per slide of each sample and counting at low power lens (magnification, 200 × ) (n = 5 per group; *P <* *.05 vs Sham + NS, #*P *<* *.05 vs AMI + NS). (C) The protein content of VEGF‐A in the heart of mice was analysed by ELISA (n = 5 per group; *P <* *.05 vs Sham + NS, # *P *<* *.05 vs AMI + NS). (D) The mRNA expressions of nucleolin and VEGF‐A in the heart of mice were analysed by real‐time qPCR (n = 5 per group; *P <* *.05 vs Sham + NS, # *P *<* *.05 vs AMI + NS). (E) The protein expression of nucleolin was detected by Western blotting, the representative blotting images (upper) and analysis of results (nether) (n = 5 per group; *P <* *.05 vs Sham + NS, # *P *<* *.05 vs AMI + NS)

Angiogenesis was proved to be beneficial to improve the ischaemic myocardial injury.^26^ So the expression of angiogenic markers (CD31, VEGF‐A) in the ischaemic myocardium was detected. It was shown that both the MVD (CD31+ endothelial cells) and VEGF‐A expressions (mRNA and protein) of ischaemic myocardium in HSYA‐treated AMI mice were substantially elevated than those of saline‐treated AMI mice (*P* < .05, Figure 3A–D). In our previous study, nucleolin was proved to play a pro‐angiogenic role during the recovery of heat‐denatured dermis through enhancing the production of VEGF‐A.^20^ So in this study, the expression of nucleolin was also detected, and we found that both the mRNA and protein expressions of nucleolin were significantly increased in the myocardium of AMI mice compared with those of the sham‐operated controls, whereas HSYA further enhanced the mRNA and protein expressions of nucleolin in the myocardium of AMI mice (*P* < .05, Figure 3C,D). Taken together, these data suggested that HSYA treatment could protect against ischaemia‐induced cardiac dysfunction, which might be related to the promotion of angiogenesis and up‐regulation of nucleolin expression.

### 3.4 HSYA promoted the migration and tube formation of HUVECs

**Figure 4:**
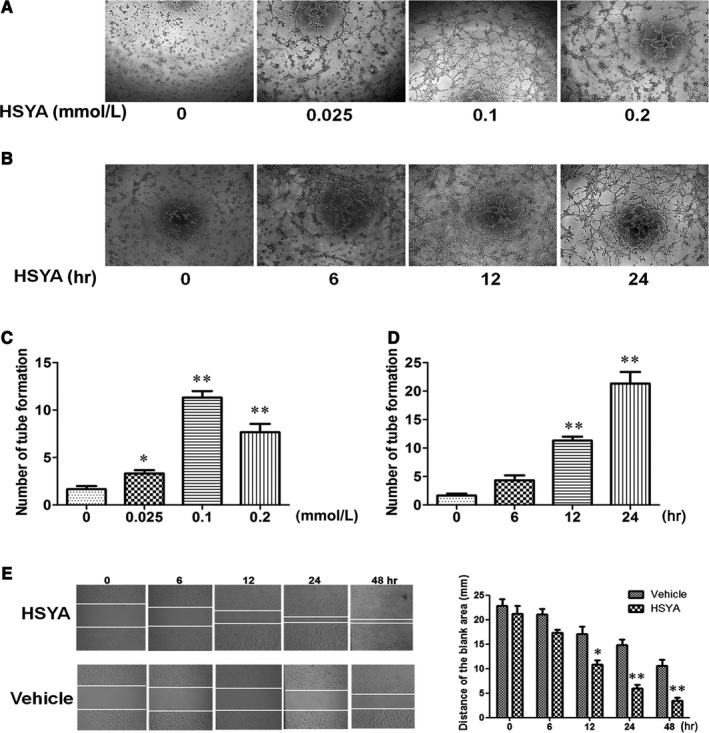
Hydroxysafflor Yellow A promoted the migration and tube formation of HUVECs. A and C, Effect of different concentrations of HSYA on the tube formation of HUVECs (magnification, ×100). Different concentrations (0, 0.025, 0.1, 0.2 mmol/L) of HSYA were treated on the HUVECs for 12 hours, the representative images of tube formation on Matrigel (A) and the analysis of results (C) (n = 3 per group; *P <* *.05, **P <* *.01 vs 0 mmol/L). B and D, Effect of treatment with 0.1 mmol/L HSYA for different time (0, 6, 12, 24 hours) on the tube formation of HUVECs (magnification, ×100). 0.1 mmol/L HSYA were treated on the HUVECs for 6, 12 and 24 hours, the representative images of tube formation on Matrigel (B) and the analysis of results (D) (n = 3 per group; *P <* *.05, **P <* *.01 vs 0 hour). E and F, Effect of treatment with 0.1 mmol/L HSYA or equal volume of vehicle for different time (0, 6, 12, 24, 48 hours) on the migration of HUVECs, the representative images of scratch wound‐healing assay (E) and the analysis of results (F) (n = 3 per group; *P <* *.05, **P <* *.01 vs Vehicle) (magnification, ×100)

As the organization of individual endothelial cells into a 3‐dimensional tube‐like structure was proved to be the initial process of angiogenesis, the effect of HSYA on the formation of capillary‐like tube structures was detected by seeding the HUVECs on the basement membrane matrix (Matrigel; BD Bioscience). As shown in Figure 4A,B, different concentrations of HSYA were treated on the HUVECs for different time, and 0.1 mmol/L HSYA most significantly promoted the tube formation of HUVECs in a time‐dependent manner, which was most obvious at 24 hours (Figure 4C,D, *P* < .05). Next, scratch wound‐healing assay was performed to detect the effect of HSYA on the migration of HUVECs. As shown in Figure 4E, 0.1 mmol/L HSYA was treated on the HUVECs for 6, 12, 24 and 48 hours, the distance of blank area gradually narrowed down with the extension of culture time, which was significantly lower at 12, 24 and 48 hours than that of initial condition (Figure 4F, *P* < .05). Thus, HSYA exerts obvious pro‐angiogenic effect via promoting the migration and tube formation of HUVECs.

### 3.5 HSYA elevated the expressions of nucleolin, VEGF‐A and MMP‐9 in HUVECs

**Figure 5:**
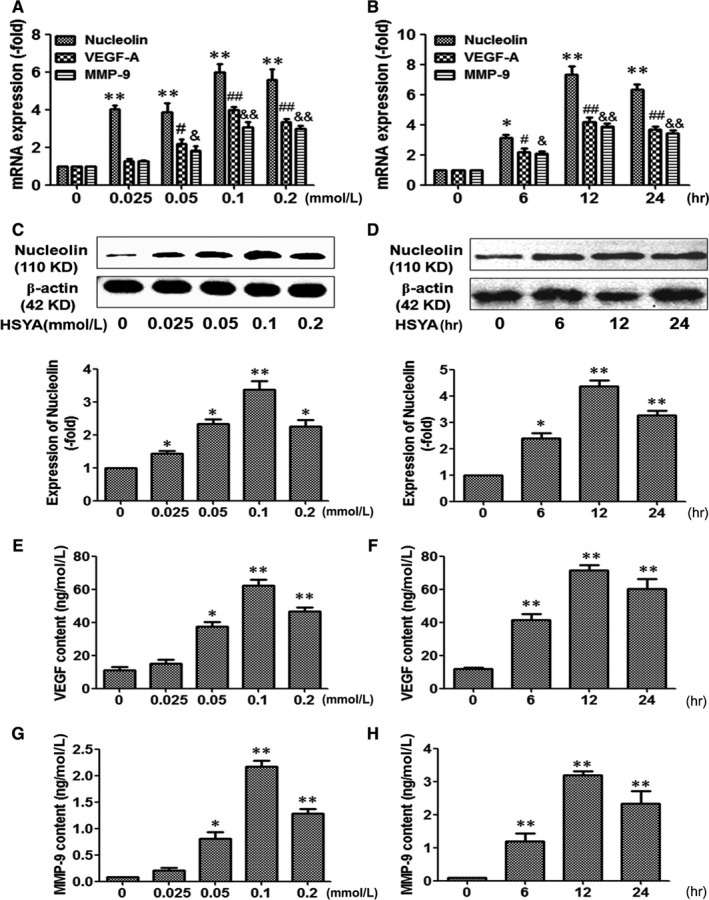
Hydroxysafflor Yellow A elevated the expressions of nucleolin, VEGF‐A and MMP‐9 in HUVECs. (A) Real‐time PCR analysis demonstrated the effect of different concentrations (0, 0.025, 0.1, 0.2 mmol/L) of HSYA on the mRNA expressions of nucleolin, VEGF‐A and MMP‐9 in HUVECs (n = 3 per group; **P <* *.01, #*P *<* *.05, &*P *<* *.05 vs 0 mmol/L). (B) Real‐time PCR analysis demonstrated the effect of treatment with 0.1 mmol/L HSYA for different time (0, 6, 12, 24 hours) on the mRNA expressions of nucleolin, VEGF‐A and MMP‐9 in HUVECs (n = 3 per group; *P <* *.05, **P <* *.01, #*P *<* *.05, &*P *<* *.05 vs 0 hour). (C) Western blotting analysis demonstrated the effect of different concentrations (0, 0.025, 0.1, 0.2 mmol/L) of HSYA on the protein expression of nucleolin (n = 3 per group; *P <* *.05, **P <* *.01 vs 0 mmol/L). (D) Western blotting analysis demonstrated the effect of treatment with 0.1 mmol/L HSYA for different time (0, 6, 12, 24 hours) on the protein expression of nucleolin (n = 3 per group; *P <* *.05, **P <* *.01 vs 0 hour). (E) and (G) ELISA analysis demonstrated the effect of different concentrations (0, 0.025, 0.1, 0.2 mmol/L) of HSYA on the protein content of VEGF‐A (E) and MMP‐9 (G) (n = 3 per group; *P <* *.05, **P <* *.01 vs 0 mmol/L). (F) and (H) ELISA analysis demonstrated the effect of treatment with 0.1 mmol/L HSYA for different time (0, 6, 12, 24 hours) on the protein content of VEGF‐A (F) and MMP‐9 (H) (n = 3 per group; *P <* *.05, **P <* *.01 vs 0 hour)

Our previous study proved that nucleolin could promote the angiogenesis through increasing the expressions of several pro‐angiogenic factors, especially VEGF‐A and MMP‐9. Therefore, the effect of HSYA on the expressions of nucleolin, VEGF‐A and MMP‐9 in HUVECs was detected. As shown in Figure 5, 0.025, 0.05, 0.1 and 0.2 mmol/L HSYA elevated the mRNA and protein expressions of nucleolin, VEGF‐A and MMP‐9, which were obviously higher in 0.1 and 0.2 mmol/L HSYA‐treated HUVECs, but no significant difference was found between 0.1 mmol/L and 0.2 mmol/L HSYA‐treated HUVECs (Figure 5A,C,E,G). In addition, 0.1 mM HSYA enhanced the mRNA and protein expressions of nucleolin, VEGF‐A and MMP‐9 with the extension of culture time, which were highest at 12 hours (Figure 5B,D,F,H). Taken together, HSYA could elevate the expressions of nucleolin, VEGF‐A and MMP‐9 in HUVECs in a dose‐ and time‐dependent manner.

### 3.6 Nucleolin was involved in HSYA‐promoted tube formation of HUVECs

**Figure 6:**
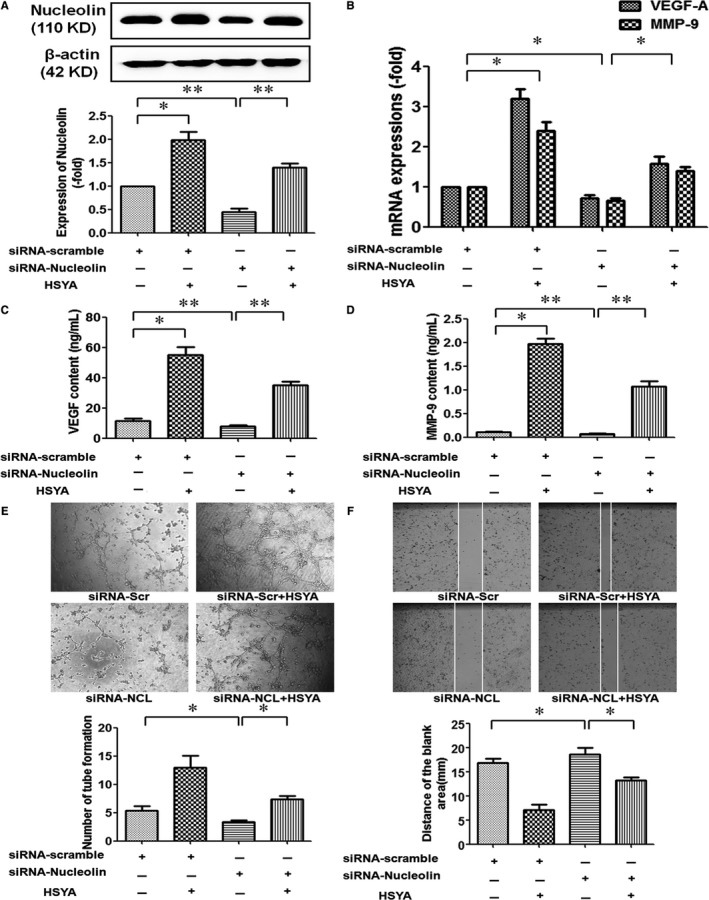
Nucleolin was involved in HSYA‐promoted tube formation of HUVECs. (A) Western blotting analysis demonstrated that HSYA partly abrogated siRNA‐nucleolin‐down‐regulated protein expression of nucleolin in HUVECs. HUVECs were transfected with siRNA‐scramble or siRNA‐nucleolin for 48 hours and then treated with or without 0.1 mmol/L HSYA for 24 hours (n = 3 per group; *P <* *.05, **P <* *.01). (B) RT‐PCR analysis demonstrated the effect of nucleolin ablation on the expressions of VEGF‐A and MMP‐9 mRNA in HUVECs treated by HSYA (n = 3 per group; *P <* *.05). (C) and (D) ELISA analysis demonstrated the effect of nucleolin ablation on the protein content of VEGF‐A and MMP‐9 in the culture supernatant of HUVECs treated by 0.1 mmol/L HSYA (n = 3 per group; *P <* *.05, **P <* *.01). (E) and (F) Tube formation on Matrigel assay and scratch wound‐healing assay demonstrated the effect of nucleolin ablation on the tube formation (E) and migration (F) of HUVECs, the representative images (upper) and the analysis of results (nether) (n = 3 per group; *P <* *.05) (magnification, ×100)

As we have proved above, HSYA not only promoted the tube formation of HUVECs, but also elevated the expressions of nucleolin and pro‐angiogenic factors in the HUVECs, then whether nucleolin mediated the pro‐angiogenic effect of HSYA? Next, HUVECs were transfected by siRNA‐NCL and corresponding siRNA‐scramble, as shown in Figure 6A, siRNA‐NCL evidently decreased the protein expression of nucleolin, whereas HSYA effectively up‐regulated the protein expression of nucleolin (*P* < .05). Accordingly, HSYA obviously elevated the mRNA expressions of VEGF‐A and MMP‐9, VEGF‐A content in the culture medium of HUVECs treated by siRNA‐scramble, while siRNA‐NCL significantly abrogate the effect mentioned above of HSYA (Figure 6B,C, *P* < .05). Furthermore, HUVECs transfected by siRNA‐NCL and siRNA‐scramble were seeded on Matrigel, it was found that tube‐like structures appeared on Matrigel at 24 hours after HSYA treatment, and the numbers of tube formation were markedly higher in HSYA‐treated HUVECs than that of HUVECs treated by siRNA‐scramble alone, whereas down‐regulation of nucleolin expression by siRNA‐NCL substantially reversed the effect of HSYA (Figure 6D, *P* < .05). These results suggested that nucleolin was involved in HSYA‐promoted tube formation of HUVECs.

### 3.7 Nucleolin binded to the VEGF‐A and MMP‐9 mRNA and up‐regulated the mRNA expressions of VEGF‐A and MMP‐9 in the HUVECs

**Figure 7:**
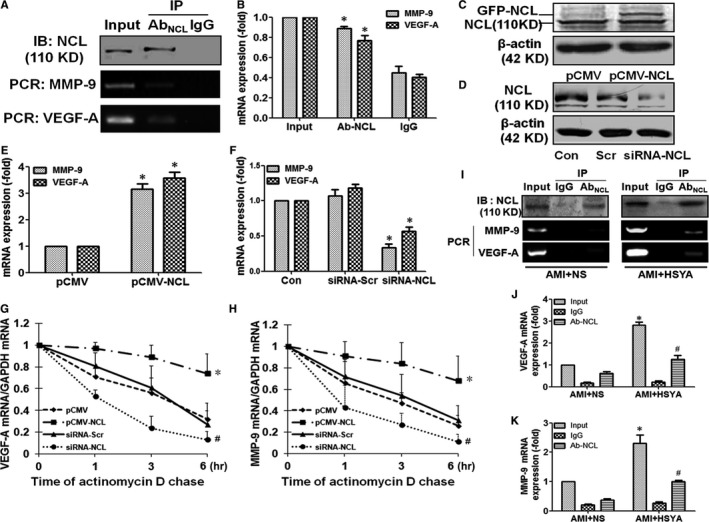
Nucleolin binded to VEGF‐A and MMP‐9 mRNA and up‐regulated the expressions of VEGF‐A and MMP‐9 mRNA through enhancing their stability. (A) and (B) IP‐RT‐PCR analysis demonstrated that nucleolin binded to VEGF‐A and MMP‐9 mRNA. Nucleolin in the HUVECs was precipitated by nucleolin antibody and magnetic A/G magnetic beads, Western blotting was performed to identify the precipitation efficiency of nucleolin, and the mRNA expressions of MMP‐9 and VEGF‐A were detected in the extracted from the magnetic beads, the representative images (A) and the analysis of results (B) (n = 3 per group; *P <* *.05 vs IgG). (C) and (D) Western blotting demonstrated the effect of transfection with pCMV‐NCL (C) or siRNA‐NCL (D) on the protein expression of nucleolin in the HUVECs. E and F, RT‐PCR analysis demonstrated the effect of transfection with pCMV‐NCL (E) or siRNA‐NCL (F) on the expressions of VEGF‐A and MMP‐9 mRNA (n = 3 per group; *P <* *.05 vs pCMV‐GFP (pCMV) or siRNA‐scramble (siRNA‐Scr). (G) and (H) Effect of up‐regulation and down‐regulation of nucleolin expression on the stability of VEGF‐A (G) and MMP‐9 (H) mRNA. HUVECs were transfected with pCMV, pCMV‐NCL, siRNA‐Scr or siRNA‐NCL and then incubated with actinomycin D (5 mg/mL) for various time (0, 1, 3, 6 hours), and the mRNA levels of VEGF‐A and MMP‐9 were determined by real‐time PCR, respectively (n = 3 per group; *P <* *.05 vs pCMV; # *P *<* *.05 vs siRNA‐Scr). I–K, IP‐RT‐PCR assay demonstrated the interaction between nucleolin and VEGF‐A, nucleolin and MMP‐9 in the myocardial tissue of AMI mice and HSYA‐treated AMI mice. Nucleolin in the myocardial tissue was precipitated by nucleolin antibody and magnetic A/G beads, Western blotting was performed to identify the precipitation efficiency of nucleolin, the mRNA expressions of VEGF‐A and MMP‐9 were detected in the extracted from magnetic beads by RT‐PCR, and the representative images (I) and analysis of VEGF‐A (J) and MMP‐9 (K) mRNA were shown (n = 3 per group; *P <* *.05, #*P *<* *.05 vs AMI + NS)

Nucleolin, an important nucleolar RNA‐binding protein, had been proved to be able to regulate the mRNA expressions of target genes which had nucleolin‐binding element ((U/G)CCCG(A/G)). Therefore, we firstly examined whether nucleolin could bind to VEGF‐A and MMP‐9 by protein‐RNA coimmunoprecipitation. As shown in Figure 7A, nucleolin in the HUVECs was successfully precipitated through nucleolin antibody and magnetic A/G magnetic beads, and the mRNA expressions of MMP‐9 and VEGF‐A were detectable in the extracted from the magnetic beads, suggesting that nucleolin could bind to MMP‐9 and VEGF‐A mRNA. In addition, 100/Ct value was used to evaluate the mRNA expression. It was shown that the expressions of MMP‐9 and VEGF‐A mRNA in the lysates of HUVECs precipitated with antinucleolin antibody were equal to the those HUVECs without antinucleolin antibody treatment, but they were significantly higher than those HUVECs treated by IgG (Figure 7B, *P* < .05). These results demonstrated that nucleolin could interact with the MMP‐9 and VEGF‐A mRNA. Next, nucleolin was successfully overexpressed in the pCMV‐NCL‐transfected HUVECs, while siRNA‐NCL obviously down‐regulated the expression of nucleolin protein (Figure 7C,D). We found that the expressions of MMP‐9 and VEGF‐A mRNA were significantly up‐regulated by overexpression of nucleolin, whereas NCL‐siRNA displayed contrary effect on the expressions of MMP‐9 and VEGF‐A mRNA in the HUVECs (Figure 7E,F, *P* < .05).

### 3.8 Nucleolin up‐regulated the mRNA expressions of VEGF‐A and MMP‐9 through enhancing the stability of VEGF‐A and MMP‐9 mRNA

Our previous studies had proved that nucleolin regulated the mRNA expressions of target genes through increasing the stability of their mRNA. Thus, we further investigated the effect of nucleolin on the stability of VEGF‐A and MMP‐9 mRNA. It was shown that both the degradation rates of VEGF‐A and MMP‐9 mRNA in the pCMV‐GFP‐NCL‐transfected HUVECs were significantly decreased as compared with negative control plasmid‐transfected HUVECs, whereas the degradation rates of VEGF‐A and MMP‐9 mRNA in the NCL‐siRNA‐transfected HUVECs were prominently lower than those in the scramble siRNA‐transfected HUVECs (Figure 7G,H, *P* < .05). These data demonstrated that nucleolin up‐regulated the expressions of VEGF‐A and MMP‐9 mRNA through enhancing their stability.

To further identify that the regulation of nucleolin on the mRNA of VEGF‐A and MMP‐9 was involved in the pro‐angiogenic effect of HSYA, the interaction between nucleolin and mRNA of VEGF‐A and MMP‐9 in HSYA‐treated AMI mice was determined by IP‐RT‐PCR. We found that nucleolin in the heart tissues of AMI mice and HSYA‐treated AMI mice was successfully precipitated through protein A/G magnetic beads, and the expressions of MMP‐9 and VEGF‐A mRNA were detectable in the extracted from the magnetic beads, suggesting that nucleolin could bind to the MMP‐9 and VEGF‐A mRNA in the heart of saline‐ and HSYA‐treated AMI mice (Figure 7I). Moreover, both the expressions of MMP‐9 and VEGF‐A mRNA were relatively weak in the extract of nucleolin antibody‐precipitated protein from the heart tissue of AMI mice, which were prominently increased by HSYA treatment (Figure 7J,K, *P* < .05). These results suggested that HSYA treatment could enhance the interaction between nucleolin and mRNA of VEGF‐A and MMP‐9 in the myocardium of AMI mice.

## 4 DISCUSSION

Angiogenesis is a vital and sequential process in the growth and development as well as wound healing. It is characterized by that successively smaller blood vessels sprout from existing ones to form networks of capillaries.^27^ Firstly, proteolytic enzymes from the endothelial cells degrade the surrounding basement membrane, which induces the invasion of endothelial sprouts into the extracellular matrix and initiates the sprout formation, followed by the proliferation and migration of endothelial cells. Subsequently, the migrating endothelial cells form tube‐like structures which further fuse and prune to primitive vessel‐like networks and finally yield hierarchically organized and functional vessels.^5^, ^28^ Therapeutic angiogenesis is firstly proposed to describe the induction or stimulation of neovascularization for the treatment or prevention of pathological situations characterized by local hypovascularity.^29^ However, excessive angiogenesis may result in different diseases such as cancer, atherosclerosis, rheumatoid arthritis and endometriosis.^30^, ^31^ Therefore, therapeutic angiogenesis should aim to achieve effective and moderate angiogenesis.^32^

For a long time, different monomeric compounds purified from Chinese herbs such as curcumin, HSYA and artesunate have exhibited strong pro‐angiogenic or antiangiogenic effect under different pathological conditions.^14^, ^33^, ^34^, ^35^ Moreover, these monomeric compounds show little toxicity on humans, which makes them easily translatable approaches to treat angiogenesis‐related diseases. Among these monomeric compounds, the bidirectional regulation of angiogenesis by HSYA is remarkably attractive. HSYA is most well known for its anticoagulant activity and has been long used in the treatment of myocardial ischaemia. Its antiangiogenic effect was firstly found on the chick embryo chorioallantoic membrane through inhibiting the mRNA expressions of bFGF, VEGF and VEGF‐F (flt‐1).^36^ Lately, Chen et al^12^ found that HSYA promoted angiogenesis via the angiopoietin 1/tie‐2 signalling pathway using HUVECs in vitro and a mouse hindlimb ischaemia model in vivo. Wei et al.^14^ found that HSYA could promote myocardial neovascularization and improve the cardiac function of AMI mice, which attributed to improve the endothelial progenitor cells function through the HO‐1/VEGF‐A/SDF‐1α signalling cascade. In the present study, we found that HSYA could ameliorate ischaemia‐induced cardiac dysfunction, diminish the myocardial infarct size, alleviate the myocardial injury and finally improve the prognosis of myocardial ischaemia. Meanwhile, HSYA promoted the angiogenesis in the ischaemic myocardium in vivo and migration and tube formation of HUVECs in vitro. These results provided line of evidence indicating that HSYA could protect against ischaemic cardiac dysfunction, which might be related to promote the angiogenesis in ischaemic myocardium. However, the underlying mechanisms involved in the pro‐angiogenic effect of HSYA remain largely unknown.

Angiogenesis is a pro‐angiogenic factor (mainly VEGF, bFGF, MMP, etc) and antiangiogenic factors (mainly angiostatin, endostatin, thrombospondin‐1, etc) coregulated and balanced process. Among these factors, VEGF‐A and MMP‐9 are considered as important regulators of blood vessel formation, which can promote the proliferation, migration and tube‐like structure formation of vascular endothelial cells. As a result, they are considered as potential targets for angiogenesis‐related diseases.^37^, ^38^ In the present study, we found that HSYA significantly increased the VEGF‐A and MMP‐9 mRNA and protein expressions in the ischaemic myocardium, which might contribute to the pro‐angiogenic effect of HSYA. These results were partly similar to those Wei et al^14^ had reported. However, how HSYA mediated the expression of VEGF‐A and MMP‐9 had not yet been fully elucidated.

In our previous study, nucleolin which was a multifunctional phosphoprotein abundantly expressed in the nucleolus, nucleus, cytoplasm and cytomembrane was found to be able to promote the angiogenesis during the recovery of heat‐denatured HUVECs through up‐regulation the expression of VEGF.^20^, ^36^ Cell surface nucleolin is even proved to be a specific marker of angiogenic endothelial cells in the vasculature.^39^ Moreover, nucleolin displayed remarkably cardioprotective, antiapoptotic and tumour inhibitory effects, etc.^18^, ^23^, ^40^, ^41^ In the present study, we found that HSYA dominantly increased the nucleolin expression in the ischaemic myocardium and HUVECs. In addition, down‐regulating the expression of nucleolin in HUVECs abrogated HSYA‐promoted tube formation. These results suggested that nucleolin might be involved in the cardioprotective and pro‐angiogenic effect of HSYA.

As a DNA‐, RNA‐ and protein‐binding protein, nucleolin regulated the expressions of target genes through binding them with the nucleolin recognition element and further post‐transcriptionally regulated their expressions.^17^, ^18^, ^19^ Both VEGF‐A and MMP‐9 which are 2 important pro‐angiogenic factors are found to have nucleolin RNA‐binding element. In the present study, protein‐RNA coimmunoprecipitation was performed and showed that nucleolin binded to the VEGF‐A and MMP‐9 mRNA in the HUVECs, the heart of AMI mice and HSYA‐treated AMI mice. Furthermore, down‐regulation and up‐regulation of nucleolin expression increased and decreased the expressions of VEGF‐A and MMP‐9 mRNA in the HUVECs, respectively. These data indicated that nucleolin mediated the pro‐angiogenic effect of HSYA through up‐regulating the expressions of VEGF‐A and MMP‐9.

Besides the transcriptional regulation, mRNA stability also regulated the protein expressions of VEGF‐A and MMP‐9.^42^, ^43^, ^44^, ^45^ Carraway KR et al. found that KHSRP stabilized the mRNA of VEGF and increased its expression under hypoxia/hypoglycaemia conditions.^42^ Morrison et al^43^ showed that Rac2–Myosin IIA interaction in the macrophages enhanced VEGF‐A mRNA stability and promoted ischaemia‐induced arteriogenesis. Moreover, Liu WH et al. demonstrated that CIL‐102 reduced MMP‐9 expression and invasion and migration of K562 cells through simultaneous suppression of MMP‐9 genetic transcription and mRNA stability.^44^ Hwang et al^45^ proved that Kalopanaxsaponin A inhibited the invasiveness of oral cancer by reducing MMP‐9 mRNA stability and expression. The present study demonstrated that overexpression of nucleolin enhanced that stability of VEGF‐A and MMP‐9 mRNA, vice versa. Additionally, both the expressions of MMP‐9 and VEGF‐A mRNA were relatively weak in the extract of antinucleolin antibody‐precipitated protein from the heart tissue of AMI mice, which were prominently increased by HSYA treatment. These results suggested that HSYA treatment could enhance the interaction between nucleolin and mRNA of VEGF‐A and MMP‐9 in the myocardium of AMI mice, which further enhanced the stability of VEGF‐A and MMP‐9 mRNA and finally increased their expressions.

It is also worthy to be noted that HSYA exhibited obvious antiangiogenesis on different cancer models. Besides what we had mentioned above, the inhibitory effect of HSYA on the abnormal proliferation of HUVECs and angiogenesis was reported by Wang et al.^46^ Yang et al.^47^ found that HSYA inhibited the angiogenesis of hepatocellular carcinoma through blocking ERK/MAPK and NF‐κB signalling pathway in H22 tumour‐bearing mice. Furthermore, the antiangiogenic effect of HSYA was found in the transplantation tumour of gastric adenocarcinoma cell line BGC‐823‐induced nude mice.^48^ We speculated that there may be a switch point for HSYA to balance angiogenesis. Nevertheless, the on‐off switch for the effect of HSYA on the angiogenesis was still unknown, which could not be ignored and should be more focused on in the further study.

In summary, the present study showed that HSYA could ameliorate ischaemia‐induced cardiac dysfunction, alleviate ischaemia‐induced myocardial injury, increase the expression of nucleolin and promote the angiogenesis in the ischaemic myocardium. In addition, nucleolin mediated pro‐angiogenic effect of HSYA through post‐transcriptional regulation of VEGF‐A and MMP‐9 expression. The pro‐angiogenic effect of HSYA made it a promising option for various ischaemic diseases. Meanwhile, the antiangiogenic effect of HSYA on neovascular disease and cancer should also be focused on. Furthermore, further studies are needed to elucidate the underlying mechanism involved in the bidirectional effect of HSYA on angiogenesis.

## CONFLICT OF INTEREST

The authors declare that they have no conflict of interest.

## References

[1] Balakumar P , Maung‐U K , Jagadeesh G . Prevalence and prevention of cardiovascular disease and diabetes mellitus. Pharmacol Res. 2016;113:600‐609. 27697647 10.1016/j.phrs.2016.09.040

[2] Naqvi SY , Klein J , Saha T , et al. Comparison of percutaneous coronary intervention versus coronary artery bypass grafting for unprotected left main coronary artery disease. Am J Cardiol. 2017;119:520‐527. 28012553 10.1016/j.amjcard.2016.11.003

[3] Caggegi A , Capodanno D , Capranzano P , et al. Comparison of one‐year outcomes of percutaneous coronary intervention versus coronary artery bypass grafting in patients with unprotected left main coronary artery disease and acute coronary syndromes (from the CUSTOMIZE Registry). Am J Cardiol. 2011;108:355‐359. 21545992 10.1016/j.amjcard.2011.03.050

[4] Eming SA , Brachvogel B , Odorisio T , et al. Regulation of angiogenesis: wound healing as a model. Prog Histochem Cytochem. 2007;42:115‐170. 17980716 10.1016/j.proghi.2007.06.001

[5] Breier G , Damert A , Plate KH , et al. Angiogenesis in embryos and ischemic diseases. Thromb Haemost. 1997;78:678‐683. 9198238

[6] Decker CG , Wang Y , Paluck SJ , et al. Fibroblast growth factor 2 dimer with superagonist in vitro activity improves granulation tissue formation during wound healing. Biomaterials. 2016;81:157‐168. 26731578 10.1016/j.biomaterials.2015.12.003 PMC4715972

[7] Ware JA , Simons M . Angiogenesis in ischemic heart disease. Nat Med. 1997;3:158‐164. 9018233 10.1038/nm0297-158

[8] Mandic L , Traxler D , Gugerell A , et al. Molecular imaging of angiogenesis in cardiac regeneration. Curr Cardiovasc Imaging Rep. 2016;9:27. 27683600 10.1007/s12410-016-9389-6 PMC5018257

[9] Wang J , Zhou J , Wang Y , et al. Qiliqiangxin protects against anoxic injury in cardiac microvascular endothelial cells via NRG‐1/ErbB‐PI3K/Akt/mTOR pathway. J Cell Mol Med 2017;21:1905‐1914. 28271613 10.1111/jcmm.13111 PMC5571527

[10] Xu X , Guo Y , Zhao J , et al. Hydroxysafflor Yellow A Inhibits LPS‐induced NLRP3 inflammasome activation via binding to xanthine oxidase in mouse RAW264.7 macrophages. Mediators Inflamm. 2016;2016:8172706. 27433030 10.1155/2016/8172706 PMC4940575

[11] Wang Y , Zhang C , Peng W , et al. Hydroxysafflor Yellow A exerts antioxidant effects in a rat model of traumatic brain injury. Mol Med Rep. 2016;14:3690‐3696. 27599591 10.3892/mmr.2016.5720 PMC5042747

[12] Chen T , Chen N , Pang N , et al. Hydroxysafflor Yellow A promotes angiogenesis via the angiopoietin 1/Tie‐2 signaling pathway. J Vasc Res. 2016;53:245‐254. 27894114 10.1159/000452408

[13] Zhang Y , Song L , Pan R , et al. Hydroxysafflor Yellow A alleviates lipopolysaccharide‐induced acute respiratory distress syndrome in mice. Biol Pharm Bull. 2017;40:135‐144. 28154251 10.1248/bpb.b16-00329

[14] Wei G , Yin Y , Duan J , et al. Hydroxysafflor Yellow A promotes neovascularization and cardiac function recovery through HO‐1/VEGF‐A/SDF‐1alpha cascade. Biomed Pharmacother. 2017;88:409‐420. 28122306 10.1016/j.biopha.2017.01.074

[15] Abdelmohsen K , Gorospe M . RNA‐binding protein nucleolin in disease. RNA Biol. 2012;9:799‐808. 22617883 10.4161/rna.19718 PMC3495746

[16] Tajrishi MM , Tuteja R , Tuteja N . Nucleolin: The most abundant multifunctional phosphoprotein of nucleolus. Commun Integr Biol. 2011;4:267‐275. 21980556 10.4161/cib.4.3.14884 PMC3187884

[17] Serin G , Joseph G , Ghisolfi L , et al. Two RNA‐binding domains determine the RNA‐binding specificity of nucleolin. J Biol Chem. 1997;272:13109‐13116. 9148924 10.1074/jbc.272.20.13109

[18] Jiang B , Liang P , Wang K , et al. Nucleolin involved in myocardial ischaemic preconditioning via post‐transcriptional control of HSPA1A expression. Cardiovasc Res. 2014;102:56‐67. 24442868 10.1093/cvr/cvu006

[19] Cheng DD , Zhao HG , Yang YS , et al. GSK3beta negatively regulates HIF1alpha mRNA stability via nucleolin in the MG63 osteosarcoma cell line. Biochem Biophys Res Commun. 2014;443:598‐603. 24333432 10.1016/j.bbrc.2013.12.020

[20] Liang P , Jiang B , Lv C , et al. The expression and proangiogenic effect of nucleolin during the recovery of heat‐denatured HUVECs. Biochim Biophys Acta. 2013;1830:4500‐4512. 23726991 10.1016/j.bbagen.2013.05.027

[21] Fahling M , Steege A , Perlewitz A , et al. Role of nucleolin in posttranscriptional control of MMP‐9 expression. Biochim Biophys Acta. 2005;1731:32‐40. 16153722 10.1016/j.bbaexp.2005.08.005

[22] Zhang C , Feng Y , Qu S , et al. Resveratrol attenuates doxorubicin‐induced cardiomyocyte apoptosis in mice through SIRT1‐mediated deacetylation of p53. Cardiovasc Res. 2011;90:538‐545. 21278141 10.1093/cvr/cvr022

[23] Jiang B , Zhang B , Liang P , et al. Nucleolin protects the heart from ischaemia‐reperfusion injury by up‐regulating heat shock protein 32. Cardiovasc Res. 2013;99:92‐101. 23594402 10.1093/cvr/cvt085

[24] Wang N , Mao L , Yang L , et al. Resveratrol protects against early polymicrobial sepsis‐induced acute kidney injury through inhibiting endoplasmic reticulum stress‐activated NF‐kappaB pathway. Oncotarget. 2017;8:36449‐36461. 28430592 10.18632/oncotarget.16860 PMC5482667

[25] Wang N , Zhang D , Mao X , et al. Astragalus polysaccharides decreased the expression of PTP1B through relieving ER stress induced activation of ATF6 in a rat model of type 2 diabetes. Mol Cell Endocrinol. 2009;307:89‐98. 19524131 10.1016/j.mce.2009.03.001

[26] Marti HH , Risau W . Angiogenesis in ischemic disease. Thromb Haemost. 1999;82(Suppl. 1):44‐52. 10695485

[27] Tonnesen MG , Feng X , Clark RA . Angiogenesis in wound healing. J Investig Dermatol Symp Proc. 2000;5:40‐46. 10.1046/j.1087-0024.2000.00014.x 11147674

[28] Hansen‐Smith FM . Capillary network patterning during angiogenesis. Clin Exp Pharmacol Physiol. 2000;27:830‐835. 11022978 10.1046/j.1440-1681.2000.03341.x

[29] Hockel M , Schlenger K , Doctrow S , et al. Therapeutic angiogenesis. Arch Surg. 1993;128:423‐429. 7681277 10.1001/archsurg.1993.01420160061009

[30] Healy DL , Rogers PA , Hii L , et al. Angiogenesis: a new theory for endometriosis. Hum Reprod Update. 1998;4:736‐740. 10027628 10.1093/humupd/4.5.736

[31] Griffioen AW , Molema G . Angiogenesis: potentials for pharmacologic intervention in the treatment of cancer, cardiovascular diseases, and chronic inflammation. Pharmacol Rev. 2000;52:237‐268. 10835101

[32] Kornowski R , Fuchs S , Leon MB , et al. Delivery strategies to achieve therapeutic myocardial angiogenesis. Circulation. 2000;101:454‐458. 10653839 10.1161/01.cir.101.4.454

[33] Wang S , Zheng Z , Weng Y , et al. Angiogenesis and anti‐angiogenesis activity of Chinese medicinal herbal extracts. Life Sci. 2004;74:2467‐2478. 15010258 10.1016/j.lfs.2003.03.005

[34] Zhou HJ , Wang WQ , Wu GD , et al. Artesunate inhibits angiogenesis and downregulates vascular endothelial growth factor expression in chronic myeloid leukemia K562 cells. Vascul Pharmacol. 2007;47:131‐138. 17581794 10.1016/j.vph.2007.05.002

[35] Bai Y , Wang W , Sun G , et al. Curcumin inhibits angiogenesis by up‐regulation of microRNA‐1275 and microRNA‐1246: a promising therapy for treatment of corneal neovascularization. Cell Prolif. 2016;49:751‐762. 27625050 10.1111/cpr.12289 PMC6496891

[36] Qian Z , Xin N , Yan Y , et al. Research on the mechanism of Hydroxysafflor Yellow A in inhibiting angiogenesis. J Beijing Univ Tradit Chinese Med. 2004;3:25‐29.

[37] Chekhonin VP , Shein SA , Korchagina AA , et al. VEGF in tumor progression and targeted therapy. Curr Cancer Drug Targets. 2013;13:423‐443. 23167597 10.2174/15680096113139990074

[38] Mac GF , Qutub AA , Annex BH , et al. Systems biology of pro‐angiogenic therapies targeting the VEGF system. Wiley Interdiscip Rev Syst Biol Med. 2010;2:694‐707. 20890966 10.1002/wsbm.92 PMC2990677

[39] Christian S , Pilch J , Akerman ME , et al. Nucleolin expressed at the cell surface is a marker of endothelial cells in angiogenic blood vessels. J Cell Biol. 2003;163:871‐878. 14638862 10.1083/jcb.200304132 PMC2173679

[40] Abdelmohsen K , Tominaga K , Lee EK , et al. Enhanced translation by Nucleolin via G‐rich elements in coding and non‐coding regions of target mRNAs. Nucleic Acids Res. 2011;39:8513‐8530. 21737422 10.1093/nar/gkr488 PMC3201861

[41] Willimott S , Wagner SD . Post‐transcriptional and post‐translational regulation of Bcl2. Biochem Soc Trans. 2010;38:1571‐1575. 21118128 10.1042/BST0381571

[42] Carraway KR , Johnson EM , Kauffmann TC , Fry NJ , Mansfield KD . Hypoxia and hypoglycemia synergistically regulate mRNA stability. RNA Biol 2017;14:938‐951. 28362162 10.1080/15476286.2017.1311456 PMC5546718

[43] Morrison AR , Yarovinsky TO , Young BD , et al. Chemokine‐coupled beta2 integrin‐induced macrophage Rac2‐Myosin IIA interaction regulates VEGF‐A mRNA stability and arteriogenesis. J Exp Med. 2014;211:1957‐1968. 25180062 10.1084/jem.20132130 PMC4172219

[44] Liu WH , Chen YL , Chang LS . CIL‐102 induces matrix metalloproteinase‐2 (MMP‐2)/MMP‐9 down‐regulation via simultaneous suppression of genetic transcription and mRNA stability. Int J Biochem Cell Biol. 2012;44:2212‐2222. 22964005 10.1016/j.biocel.2012.08.021

[45] Hwang YS , Park KK , Chung WY . Kalopanaxsaponin A inhibits the invasion of human oral squamous cell carcinoma by reducing metalloproteinase‐9 mRNA stability and protein trafficking. Biol Pharm Bull. 2012;35:289‐300. 22382313 10.1248/bpb.35.289

[46] Wang J , Wang J , Wang X , et al. Molecular mechanism of inhibition of the abnormal proliferation of human umbilical vein endothelial cells by hydroxysafflor‐yellow A. Pharm Biol. 2016;54:1800‐1807. 26730646 10.3109/13880209.2015.1129541

[47] Yang F , Li J , Zhu J , et al. Hydroxysafflor Yellow A inhibits angiogenesis of hepatocellular carcinoma via blocking ERK/MAPK and NF‐kappaB signaling pathway in H22 tumor‐bearing mice. Eur J Pharmacol. 2015;754:105‐114. 25720342 10.1016/j.ejphar.2015.02.015

[48] Xi S , Zhang Q , Xie H , et al. [Effects of Hydroxysafflor Yellow A on blood vessel and mRNA expression with VEGF and bFGF of transplantation tumor with gastric adenocarcinoma cell line BGC‐823 in nude mice]. Zhongguo Zhong Yao Za Zhi. 2009;34:605‐610. 19526794

